# Cognitive changes in alcohol-induced psychotic disorder

**DOI:** 10.1186/s13104-017-2485-0

**Published:** 2017-04-26

**Authors:** Melany L. Hendricks, R. A. Emsley, D. G. Nel, H. B. Thornton, G. P. Jordaan

**Affiliations:** 1 Department of Psychiatry, Health Sciences Faculty, Stellenbosch University, P.O Box 241, Cape Town, 8000 South Africa; 2 Centre for Statistical Consultation, Health Sciences Faculty, Stellenbosch University, Cape Town, South Africa

**Keywords:** Alcohol, Dependence, Psychotic, Disorder, Neuropsychology, Psychometry

## Abstract

**Aims:**

This study aimed to explore the neuro-cognitive deficits of alcohol-induced psychotic disorder as compared to the cognitive deficits of uncomplicated alcohol dependence.

**Methods:**

Participants were recruited from the acute psychiatric admission wards of the Department of Psychiatry, University of Stellenbosch and Stikland and Tygerberg Academic Hospitals in the Western-Cape, South Africa. Participants who met DSM IV TR criteria (American Psychiatric Association. Diagnostic and statistical manual of mental disorders. American Psychiatric Association, Washington, DC, 2000) for Alcohol Dependence and for alcohol-induced psychotic disorder, respectively, were included. Participants who met criteria for another current DSM IV TR Axis I disorder were excluded. A structured interview was done prior to neuropsychological assessment to ascertain current mental state and to obtain relevant demographic detail and history. Neuropsychological assessments were performed and supervised by clinical psychologists at either Tygerberg or Stikland Hospital.

**Results:**

The groups were matched demographically with similar period of abstinence prior to assessment. The alcohol-induced psychotic disorder group experienced first psychotic symptoms at age 35. The results reflected statistically significant differences on tasks measuring immediate memory; recall upon delay; exaggeration of memory difficulty and abstract thinking.

**Conclusion:**

This study concurs with earlier literature that some cognitive deficits are greater in alcohol-induced psychotic disorder compared to uncomplicated alcohol dependence.

## Background

The understanding of alcohol-induced psychotic disorder (AIPD) as an illness phenomenon is becoming is increasingly important for the clinician. AIPD is often described as a “rare complication” [1] of alcohol use disorders. Despite this description, it was noted that the number of persons’ diagnosed with alcohol psychosis has escalated by four times in certain countries [2] and for patients diagnosed with AIPD there is 68% risk of re-admission [3]. There is a 37% co-morbidity of AIPD with other mental disorders [4] and a 5–30% risk that patients with AIPD will develop a chronic schizophrenia-like syndrome [5]. Patients who develop AIPD are at risk of becoming suicidal and need to be carefully monitored [3].

Recent research [1, 6] concurs with earlier findings [7] that AIPD is a distinct diagnostic entity that can be distinguished from schizophrenia. Most commonly, AIPD mostly occurs with auditory hallucinations but also delusions, often of a paranoid nature. Whilst AIPD resembles paranoid schizophrenia, it generally occurs without negative symptoms [1, 7]. The psychotic episode reportedly often lasts no more than a month [8] to 6 months [6, 9], but during that time, reality testing is impaired. The person suffering from AIPD related auditory hallucinations has no insight into the fact that hallucinations are substance-induced [10] especially during the episode.

There are inconsistent reports regarding the outcome of this disorder [2, 5]. Some authors [2, 5] noted that AIPD may become prolonged and schizophrenia-like symptoms may develop. Other authors [9, 11] noted that the outcome of AIPD is usually good if abstinence is achieved. There is some indication that AIPD is a severe psychiatric disorder with poor prognosis [4]. This study [4] however included in their sample persons who suffered from alcohol-withdrawal delirium. A fifty year follow-up study [12] concurred with the previous study [4] and found that alcohol-induced psychotic syndrome (AIPS) is associated with increased risk for premature death. This study included sample persons with delirium tremens as well as AIPD, hence the use of the term AIPS. The authors however conclude that AIPD is a severe mental illness with poor prognosis [4].

The age of onset of alcoholism reported in AIPD varied between 21.4 [2] and 29.1 years [13] with the latter study showing a significantly younger age of onset of alcoholism for AIPD patients than their non-psychotic alcohol-dependent male counterparts. There were no significant differences [13] with reference to age, education, marital status and employment between male alcoholic patients with and without a history of psychosis. In this study [13] however there was a significant difference between the number of Caucasian men diagnosed with AIPD and the number of non-Caucasian men. AIPD was diagnosed more in the Caucasian men. A previous study [4] reported that low socio-economic status, paternal mental health or alcohol related difficulties, early onset of alcohol dependence and numerous hospitalizations increase the risk for developing AIPD.

Although a number of reports [2, 14, 15] addressed the phenomenological expression of AIPD, the nuanced clinical presentation received very little scientific attention [16]. This paucity of interest is deemed unjustified as the correct diagnosis of this condition is key to accurate treatment and improved prognosis [16]. This paucity of literature highlights the lack of scientific evidence addressing the cognitive deficits of this disorder. There is some evidence suggesting that cognitive deficits as measured by the mini-mental state examination (MMSE) [17] may be greater in AIPD compared to uncomplicated AD [1].

The present introductory study compared the neuropsychological functioning of patients who were diagnosed with AIPD with the neuropsychological functioning of alcohol dependent patients in order to differentiate between these two conditions from a neuropsychological perspective. Additionally, this paper aims to expose the associated brain region which is potentially at the genesis of AIPD. Understanding the underlying pathophysiology may lead to more effective treatment of AIPD and prevent relapse, including recurrent re-admissions and possibly the development of more severe pathology.

### Neuropsychological deficits associated with alcohol use

The susceptibility to acquire neuropsychological deficits resulting from alcohol use or abuse is multifactorial [18]. In addition to alcohol use, there are several contributory elements to cognitive decline such as current age, age at which alcohol consumption started, the period of time over which alcohol was consumed, family history of alcoholism, nutrition, and conditions related to birth and pregnancy [18]. The literature concurs that increasing age and medical co-morbidity exacerbates the negative cognitive sequelae of alcohol use disorders [19]. On the other hand, there seems to be little support for the hypothesis that gender, specifically being female, is a risk factor for cognitive decline despite the fact that women appear to be more prone to alcoholism than men [18]. Chronic malnutrition, in the presence of alcohol dependence or abuse, is considered a consequence of alcohol abuse, specifically thiamine deficiency which again may lead to neurological deficits [20]. These neurological deficits, acquired through thiamine deficiency may be reversible given improved nutrition or treatment.

Alcohol misuse may cause diffuse brain damage therefore most domains of neuropsychological assessment may be affected [21]. Even alcohol-dependent patients without dementia may have mild cognitive deficits in neuropsychological functioning [21, 22]. Alcohol dependent subjects often have deficits of working memory, immediate and delayed memory as well as recognition; visual-constructive ability and verbal fluency [21].

Vocabulary is usually not affected by alcohol abuse [19, 23], but other language functions such as abstraction and comprehension were found to be impaired [19]. There is evidence showing that alcohol misuse may cause difficulties in attention and concentration [22–24]. Motor control difficulties such as impairment of gait, balance and speed were also reported in the presence of alcohol abuse [20].

Alcohol abuse is often detrimental to memory function [19, 20, 22, 25, 26]. Mild deficits in explicit memory were found in individuals who abused alcohol [20] and difficulties in short term memory and learning problems were described [27]. Chronic excessive alcohol intake was associated with difficulties in learning of new and complex verbal material [27]. Alcohol misuse harms word recognition but not the ability to identify distractor words [28]. Impairments in episodic memory, that is memory for autobiographical information, were specifically noted for persons with AD [29, 30].

A number of authors [20, 23, 26] reported poor visuospatial functioning and visuospatial processing associated with alcoholism. One author [23] asserted that patients with AD demonstrate deficiency on new tasks that involves integration and manipulation of material. Alcohol dependent persons’ performed poorer on tasks of response inhibition, executive function and attentional control [31].

Neuropsychological deficits of uncomplicated alcohol use disorders include mild cognitive deficits in executive functions [20]. Specific executive functions impaired in this population include planning, switching, correction, self-monitoring and decision-making [20]. More distinct impaired decision-making is associated with more severe alcoholism [32]. Alcohol dependent patients demonstrated deficits on tasks of rule detection, inhibition of dominant responses, coordination of dual tasks [32], and difficulties in problem-solving ability [26].

Although numerous studies elaborated on the complexity and diversity of the neuro-cognitive fall-out associated with alcohol use, there has been very little focus on AIPD. Earlier reports by Bleuler 1916 [14] generally noted that for patients suffering from AIPD; memory, attention and concentration are intact. These were based on observation, rather than neuropsychological assessments. Reporting on a retrospective review of hospital admission records, Surawics [33] noted that patients with AIPD demonstrated deficits in arithmetic, figure drawing and matching sets. These patients only occasionally exhibited fleeting memory difficulties which were associated with the duration and severity of alcohol abuse history. Both patients with schizophrenia [34] and alcohol-dependent patients with a history of hallucinations [35] have demonstrated similar deficits in source-monitoring of information. It was reported that alcohol-dependent patients with a history of hallucinations were prone to confuse imagination with reality (e.g. discriminating external versus internal source of information) [35]. Apart from these reports [33–35], we found no previous reports assessing neuropsychological differences between patients with uncomplicated AD and AIPD.

This introductory study formed part of a larger study aimed at differentiating alcohol-induced psychotic disorder (AIPD) from uncomplicated AD and Schizophrenia [1]. In particular, this study aimed to explore the neuro-cognitive deficits of AIPD as compared to the cognitive deficits of uncomplicated alcohol dependence (AD). We postulated that cognitive deficits in AIPD would be more severe than in uncomplicated AD [1].

## Methods

### Subjects

Two groups (AIPD and AD) of study participants were recruited. The AIPD group (n = 13) were recruited from the acute psychiatric admission wards of the Department of Psychiatry, University of Stellenbosch and Stikland and Tygerberg Academic Hospitals in the Western-Cape, South Africa. The AD group (n = 16) was recruited from an alcohol rehabilitation unit at Stikland Hospital.

Before the neuropsychological assessments were completed, a structured interview using the Mini International Neuropsychiatric Interview (Version 4.4) [1, 36], was performed to ascertain diagnoses according to DSM IV TR criteria. Current mental state and relevant demographic detail and history were also documented. Participants who met DSM IV TR criteria [10] for AD and for AIPD, respectively, were included. Patients who met criteria for another current DSM IV TR Axis I disorder including a history of recent other substance abuse and those with other clinically significant medical conditions, including structural brain lesions, or those who had used any psychotropic medication (excluding benzodiazepines to a maximum of 4 mg Lorazepam equivalent per day), during the 10 days prior to the study were excluded. Careful attention was therefore given to exclude patients with current alcohol-intoxication, alcohol-withdrawal or alcohol-withdrawal delirium in both the uncomplicated alcohol-dependent and AIPD groups. Patients with complicated alcohol dependence other than AIPD were also excluded from the study (e.g. patients with alcohol-induced persistent dementia and alcohol-induced persistent amnestic disorder). Patients with a history of psychotic symptoms were likewise excluded from the AD group.

### Psychological assessments

The severity of alcohol dependence questionnaire (SADQ) was used to determine the severity of alcohol dependence in both groups [37, 38]. Although self-report questionnaires are generally less reliable [40], it was demonstrated to be both valid and reliable for measuring the severity of alcohol use [41]. Although participants’ memory difficulties may influence the validity of the self-report questionnaire [41], the SADQ was found to be valid and reliable as it measures severity of alcohol dependence with reference to its essential structure [38].

We assessed the severity of psychotic symptoms by applying the positive and negative syndrome scale for schizophrenia (PANSS) [1, 39] on patients with AIPD.

### Neuropsychological assessment

Neuropsychological assessments were performed and supervised by two clinical psychologists at either Tygerberg or Stikland Hospital.

The following neuropsychological test battery was administered:

The Controlled Oral Word Association Task (COWAT) is a measure of verbal fluency through assessing the ability of a person to generate as many words as possible in a minute. Performance on the verbal fluency task involves short term immediate memory, cognitive flexibility, and initiation [42].

The Trail Making Test (TMT) A and B provides information regarding visual scanning, speed of hand eye co-ordination and information processing. The TMT B assessed the ability to alternate between different sets of stimuli (shifting) and is useful in detecting executive and cerebral dysfunction [42], mental flexibility, speed for attention, visual search, motor function and sequencing [43].

Rey Auditory Verbal Learning Test (RAVLT) trails I–V and the delayed recall was administered to assess verbal memory and learning [43].

Visual reproduction (VR) Trails I and II involves a ten second exposure to a shape, followed by immediate response. This test is considered sensitive to right hemisphere injury [40].

Rey Complex Figure-Copy measures visuospatial constructional ability and visual memory for complex visual stimuli [43]. It demonstrates planning strategies, problem-solving, perceptual motor and memory functions.

Rey 15 Item assesses the participants’ effort when completing a memory task and the extent to which memory problems are exaggerated [40, 43].

Similarities subtest of the South African Weschler Adult Intelligence Scale (Sim-SAWAIS) gauges abstract thinking by asking the patient to describe similarities between objects [40].

The Clock Drawing Test (CDT) evaluates visuospatial and constructional abilities. The clock is drawn on a blank sheet; the time on the clock should reflect 10 past 11 and the test is scored out of 10 [44].

### Statistical analysis

The two groups (AD and AIPD) were compared with respect to the continuous variables by using pooled T tests, or equivalently ANOVA. The variances of the variable Rey Fifteen Item Test (RFIT) differed between the two groups, so this T test was done by using the Satterthwaite [45] approach. The categorical/nominal variables were compared between the two AIPD groups by using contingency tables with the maximum likelihood Chi square test. The influence of highest level of education (HLOE) on the continuous responses was investigated with the analysis of covariance with HLOE as covariate. In view of the exploratory nature of the study and the small samples, we did not apply corrections for multiple comparisons.

### Ethical approval, consent and permissions

Ethical approval was obtained from the Committee for Human Research of the Faculty of Health Sciences at the University of Stellenbosch. Permission to conduct the study was obtained from the relevant Hospital authorities. All participants gave informed consent to participate in the study prior to the start thereof. Due consideration was given to the privacy, confidentiality and anonymity of the participants. The research was conducted in accordance with the prescripts of the declaration of Helsinki [46].

## Results

Table 1 represents key socio-demographic information. The two groups were found similar in terms of their socio-demographic information. The two groups were matched for age, gender (predominantly male) as well as number of days without alcohol prior to assessment. The mean age for both groups was 37. The period of abstinence prior to assessment was between 2 and 3 weeks for both groups. Both groups reported alcohol problems since approximately the age of twenty, indicating similar duration of alcohol history. The AIPD group experienced psychotic symptoms for the first time at age 35 (Table 1) suggesting a mean age of onset in the fourth decade which concurs with previous reports [1, 7]. The duration of psychotic symptoms was on average less than 2 years but often had a recurrent course starting within a month of alcohol intoxication or withdrawal.

**Table 1:** Demographic and clinical information for the alcohol-induced psychotic disorder (AIPD) and alcohol dependent (AD) groups

	AIPD (n = 13)	AD (n = 16)	Overall statistic
AGE in years, mean (SD)	37.07 (5.77)	37.50 (6.86)	F = 1.42, 27df, p = 0.86
Gender, % males	92.3 (n = 12)	87.5 (n = 14)	X^2^ = .17, 1df, p = 0.67
HLOE in years, mean (SD)	8.08 (3.84)	11.81 (2.37)	F = 2.62, 27df, p = 0.003*
Days without alcohol, mean (SD)	19.08 (11.79)	13.69 (8.07)	F = 2.13, 27df, p = 0.17
Age of onset of alcohol-related symptoms, in years, mean (SD)	20.0 (2.70)	20.5 (6.00)	F = 0.08, 27df, p = 0.78
Age of onset of psychotic symptoms in years, mean (SD)	35.7 (5.9)		
SADQ	24.83 (13.10)	37.50 (11.50)	F = 1.30, 26df, p = 0.011*

*HLOE* highest level of education

*p < 0.05 = significant

Upon initial ANOVA, the AD group had a statistically significant higher level of education (HLOE) than the AIPD group. Upon further comparative analysis with HLOE as a covariate, HLOE did not influence the findings. Even though the AD group reported more severe alcohol dependence than the AIPD group (SADQ), the difference did not reach statistical significance.

Patients with AIPD presented mainly with auditory hallucinations, delusions (with predominant persecutory themes) and anxiety in the presence of a clear consciousness. The mean scores on the PANSS scales were: 20.1 (positive scale), 16.7 (negative scale), 37.5 (general scale) and 74.3 (total scale).

Table 2 represents the comparison of results of the neuropsychological assessments between the two groups. The results reflected statistically significant differences on the RAVLT I (Immediate memory); RAVLT (recall upon delay); Rey 15-Item (exaggeration of memory difficulty) and similarities wais (abstract thinking) between the two groups.

**Table 2:** Comparison of psychometric variables for the alcohol-induced psychotic disorder (AIPD) and alcohol dependent (AD) groups

	AIPD (n = 13) mean (SD)	AD (n = 16) mean (SD)	Overall statistic (ANOVA)
COWAT % ile	42.08 (33.35)	64.50 (31.67)	F = 1.11. 27df. p = 0.08
TMT A	20.66 (17.97)	33.43 (30.16)	F = 2.81. 26df. p = 0.20
TMT B	20.64 (24.40)	40.44 (30.62)	F = 1.58. 25df. p = 0.09
RAVLT A I	18.84 (12.26)	38.68 (29.36)	F = 5.73. 27df. p = 0.03*
RAVLT A II	23.70 (26.60)	38.62 (32.30)	F = 1.47. 27df. p = 0.19
RAVLT A III	18.30 (26.55)	27.00 (32.27)	F = 1.47. 27df. p = 0.44
RAVLT A IV	33.46 (37.02)	30.31 (31.83)	F = 1.35. 27df. p = 0.81
RAVLT A V	27.50 (30.71)	43.13 (35.94)	F = 1.37. 27df. p = 0.22
RAVLT B	40.42 (37.20)	36.30 (33.24)	F = 1.25. 26df. p = 0.80
RAVLT A VI	13.96 (18.49)	38.80 (31.90)	F = 2.97. 24df. p = 0.05*
RAVLT A VII (raw score)	7.90 (2.98)	10.28 (4.95)	F = 2.76. 23df. p = 0.17
Total words	19.69 (24.60)	33.40 (33.90)	F = 1.90. 27df. p = 0.23
Words learned	48.92 (33.22)	43.44 (35.11)	F = 1.12. 27df. p = 0.20
% Recall B	48.89 (41.33)	74.98 (26.99)	F = 2.34. 24df. p = 0.06
RAVLT Err	37.00 (33.67)	44.69 (35.83)	F = 1.13. 27df. p = 0.56
RAVLT Rep	41.77 (32.42)	57.13 (22.38)	F = 2.10. 26df. p = 0.15
Recognition	41.56 (24.15)	51.21 (37.11)	F = 2.36. 21df. p = 0.50
Recall delay	74.50 (15.95)	94.44 (22.81)	F = 2.04. 24df. p = 0.08*
VR I	41.77 (35.83)	56.50 (30.45)	F = 1.38. 27df. p = 0.24
VR II	34.92 (29.80)	53.00 (33.50)	F = 1.26. 25df. p = 0.20
RCF-C	63.33 (35.05)	65.31 (30.10)	F = 1.36. 26df. p = 0.87
R15 item	10.80 (3.55)	14.14 (1.30)	F = 7.56. 22df. p = 0.004*
Sim-WAI	33.20 (33.26)	59.44 (32.69)	F = 1.04. 26df. p = 0.05*
DAC	8.90 (1.73)	9.20 (0.98)	F = 3.11. 24df. p = 0.60

*SD* standard deviation, *ANOVA* analysis of variance

* p < 0.05 = significant

## Discussion

This study demonstrated statistically significant deficit in immediate verbal memory, increased effort when completing a memory task and impaired abstract verbal reasoning skills in patients with AIPD when compared to patients with uncomplicated AD. Memory, specifically explicit memory, short term memory and learning, may be impaired by the chronic use of alcohol. Our results do not support the observations of Bleuler [14] of intact memory function for persons suffering from AIPD. On the immediate verbal memory task the AIPD group’s performance was below average while the AD group’s performance fell within the average range. Immediate memory refers to the initial phase of short term memory and theoretically it holds information before the information is registered [40]. Immediate verbal memory impairment is associated with lesions in the dominant perisylvian cortex [47]. Of interest in this regard is that neuroimaging studies suggest that the development of auditory hallucinations in schizophrenia are also associated with altered structural and functional connectivity within the perisylvian language network [48]. It may be, therefore, that similar underlying neurobiological mechanisms are involved in the genesis of auditory hallucinations in AIPD and schizophrenia.

Both groups performed above the cut-off score on the task measuring effort when completing a memory task and the extent to which memory problems are exaggerated which lends further support for more severe memory difficulties in the AIPD group compared to the AD group. On delayed verbal recall, the AIPD group performed in the below average range, while the AD group performed within the average range but the difference did not reach statistical significance. Delayed verbal recall, as measured by the RAVLT, refers to recall of words after a 2–45 min delay [40]. Impaired performance on delayed verbal recall tasks was connected to left hippocampal volume/atrophy in an Alzheimer Dementia [49] for both normal control subjects and subjects suffering from Alzheimer’s Dementia.

With reference to impairment on the task assessing abstract verbal reasoning skills, in the AIPD group, the similarities subtest is reportedly sensitive to cognitive pathology that affect verbal abstraction which is connected to left temporal and frontal involvement [40]. This may partially explain the occurrence of psychotic symptoms and supports frontal and temporal lobe involvement in AIPD [15, 50]. In addition, both groups scored within the average range for word initiation even though the AIPD group’s performance was slightly poorer than that of the AD group.

The findings concur with previously reported research suggesting cognitive impairments such as difficulty with attention and concentration are found in AIPD [23, 24]. The AIPD group performed in the below average range in tasks measuring attention and concentration and the AD group in the low average range. The difference between the two groups was not found to be statistically significant.

Both groups demonstrated ability for new learning with a positive, but fluctuating learning curve. New learning for the AIPD group fell within the below average range while new learning for the AD group was marginally better and was in the average range but the difference also did not reach statistical significance.

On tasks assessing visual memory (immediate and delayed), both groups scored within the average range with the AD group’s score better but again no statistically significant difference recorded. In terms of visuospatial assessment, both groups demonstrated average ability.

It is interesting that the AIPD group’s average of reported drinking severity (SADQ) was lower than that of the AD group. However, this may be misleading as it could be explained by recall bias in the AIPD group due to greater memory impairment, associated impairment of insight or to deliberate under reporting [40].

The study has a number of limitations. It is a cross-sectional study with a small number of participants. The participants were selected from two different clinical populations within one institution. The AD group was selected from a program that requires a certain level of education which increased the possibility of selection bias. The scope of neuropsychological exploration needs to be expanded to include all the major neuropsychological domains, such as attention and concentration, all aspects of visual and verbal memory, speech and language functioning, motor control and all aspects of executive functions. Furthermore, a detailed analysis of predisposing factors needs to be performed to rule out their association with the cognitive deficits. It therefore remains to be determined to what extent the cognitive deficits in AIPD demonstrated in this study suggests pre-existing vulnerability rather than primary effect of alcohol. These limitations, together with the fact that we did not correct for multiple comparisons, mean that our results should be regarded as preliminary.

## Conclusion

In conclusion, this study concurs with an earlier [1] that some cognitive deficits are greater in AIPD compared to uncomplicated AD. The AIPD group generally performed poorer on all tasks, but a statistically significant poorer performance was only recorded for tasks assessing immediate verbal memory, delayed verbal recall and abstract verbal reasoning abilities. These findings support the notion that several brain regions [15, 50] and possibly several neurotransmitter systems [6] are involved in the pathogenesis of AIPD. Of particular interest is the the perisylvian language network. Even though the findings of this study imply an association between AIPD and the emergence of cognitive pathology, one has to keep in mind the contribution of pre-existing factors in the development and severity of the cognitive deficits demonstrated in this study. This condition is relatively uncommon; it is often transient and generally has high levels of co-morbidity. Notwithstanding these challenges, further exploration of cognitive deficits possibly associated with other correlates should significantly improve the future understanding of AIPD and perhaps also the cognitive and neurobiological underpinnings of auditory hallucinations in other psychotic disorders.

## Abbreviations

AD: alcohol dependence
AIPD: alcohol-induced psychotic disorder
ANOVA: analysis of variance
CDT: Clock Drawing Test
COWAT: Controlled Oral Word Association Task
DSM IV TR: Diagnostic and Statistical Manual of Mental Disorder 4th Edition Text Revision
HLOE: highest level of education
PANSS: positive and negative syndrome scale for schizophrenia
RAVLT: Rey Auditory Verbal Learning Test
RFIT: Rey Fifteen Item Test
SADQ: severity of alcohol dependence questionnaire
SAWAIS: South African Weschler Adult Intelligence Scale
Sim-SAWAIS: Similarities test-South African Weschler Adult Intelligence Scale
TMT: Trail Making Test
VR: visual reproduction
